# Role of Muscarinic Receptors in Hypoalgesia Induced by Crocin in Neuropathic Pain Rats

**DOI:** 10.1155/2020/4046256

**Published:** 2020-11-25

**Authors:** Hossein Ali Safakhah, Abbas Ali Vafaei, Azin Tavasoli, Simin Jafari, Ali Ghanbari

**Affiliations:** ^1^Research Center of Physiology, Semnan University of Medical Sciences, Semnan 3519899951, Iran; ^2^Department of Physiology, Faculty of Medicine, Semnan University of Medical Sciences, Semnan 3519899951, Iran; ^3^Student Research Committee, Semnan University of Medical Sciences, Semnan 3519899951, Iran

## Abstract

**Objective:**

Crocin as an important constituent of saffron has antineuropathic pain properties; however, the exact mechanism of this effect is not known. The aim of this study was whether the hypoalgesic effect of crocin can be exerted through muscarinic receptors.

**Materials and Methods:**

In the present project, 36 male Wistar rats (200 ± 20 g) were used. Animals randomly divided into six groups (sham, neuropathy, neuropathy + crocin, neuropathy + atropine 0.5 mg/kg, neuropathy + atropine 1 mg/kg, and neuropathy + atropine 1 mg/kg + crocin). Neuropathy was induced by the chronic constriction injury (CCI) method on the sciatic nerve. Crocin and atropine was administered intraperitoneally during 14 days following the 14^th^ day after surgery. Pain response was detected every three days, two hours after each injection and 3 days following last injection. Mechanical allodynia and thermal hyperalgesia were detected using the Von Frey filaments and plantar test device, respectively.

**Results:**

CCI significantly reduced the paw withdrawal response to mechanical and thermal stimulus (*P* < 0.01 and *P* < 0.05, respectively). Crocin therapy significantly reduced mechanical allodynia and thermal hyperalgesia induced by CCI (*P* < 0.05). Atropine pretreatment significantly blocked the hypoalgesic effect of crocin (*P* < 0.05 in mechanical allodynia and *P* < 0.01 in thermal hyperalgesia). Fourteen days administration of atropine alone at a dose of 0.5 mg/kg but not 1 mg/kg significantly reduced CCI-induced mechanical allodynia at day 30 after surgery.

**Conclusion:**

Crocin significantly decreased CCI-induced neuropathic pain. The hypoalgesic effect of crocin was blocked by atropine pretreatment, which indicates an important role for muscarinic receptors in the effect of crocin.

## 1. Introduction

Neuropathic pain is one of the major challenges in medical centers throughout the world. Allodynia, hyperalgesia, and spontaneous pain are among the most current symptoms of neuropathic pain [1]. This long lasting problem occurs due to various peripheral and central mechanisms following somatosensory injuries or diseases [2, 3]. Several studies have revealed that the cholinergic system is involved in neuropathic pain. Ferrier and his colleagues reported that central muscarinic M2 receptors modulate neuropathic pain induced by traumatic nerve injury [4]. Furthermore, they showed that activation of muscarinic M2 receptors of insular cortex reduce oxaliplatin-induced neuropathic pain in male rats [4]. It has been observed that intracerebroventricular injection of oxotremorine (nonselective muscarinic agonist) leads to the antinociceptive effect [5]. Donepezil, a cholinesterase inhibitor, through spinal muscarinic receptors reduces mechanical hyperalgesia in a rat model of spinal nerve ligation [6]. According to a prior report, it seems that donepezil-induced analgesia could be due to upregulation of muscarinic M2 receptors [7]. Recently, Mendes and his colleagues showed that LASSBio-873 (a pyrazolo[3, 4]pyrrolo[3, 4]pyridine derivative), as a new muscarinic agonist, relieves spinal nerve ligation- (SNL-) induced neuropathic pain in male rats [8]. It has been reported that atropine pretreatment suppresses acetyl-L-carnitine-induced analgesia in mice and rats [9]. Numerous chemical substances are presented for treatment and management of neuropathic pain; however, none of them have been effective in relieving pain completely [4]. In addition, some medications have several side effects that limit their use [10]; so, it is important to use treatments without side effects or minimal side effects that are effective.

Psychologically, the use of natural remedies is generally more acceptable than chemicals due to fewer side effects [11–13].

Herbal medicines have been used extensively throughout the world for years [14]. Saffron is a Mediterranean plant which contains several important bioactive constituents including crocin (color of saffron), safranal (odor of saffron), and picrocrocin (taste of saffron) [15–17]. Saffron (*Crocus sativus* L), a traditional medicine, has anti-inflammatory, antinociceptive, and antioxidative properties [18, 19]. It has been shown that saffron has protective effects against hepatic disorders, asthma, depression, chronic pain, anxiety, stress, and memory deficits [20–25]. There are a few studies that show the interaction of saffron with neurotransmitter systems [22, 23]. It has been reported that in an in vitro model, saffron extract inhibits neurotransmission of the glutamatergic system in the rat brain cortex [26]. On the other hand, it has been revealed that the saffron aqueous extract increases glutamate and dopamine contents of rat brain [27]. Studies on the memory revealed interaction of saffron with the cholinergic system [28]. Another study showed that saffron inhibits acetylcholinesterase (AChE) activity through interaction with binding sites of AChE [29]. Some of beneficial effects of saffron have been attributed to crocin [30]. It has been shown that crocin has the neuroprotective effect in animal models [31, 32]. Prior research revealed that behavioral impairments (schizophrenia-like behavior) induced by ketamine were inhibited by crocin [33]. Several animal studies have shown that crocin alleviates neuropathic pain [34–36]. Crocin amplified sciatic nerve function in crush injured rats through decreasing oxidative stress [37]. There is rare information about interaction of crocin with neurotransmitter systems in neuropathic pain.

The more the mechanisms are known, the more likely to find modern therapeutic strategies to management of neuropathic pain. Considering the interaction of saffron with the cholinergic system in neuropathic pain and analgesic effect of crocin, which is an important ingredient of saffron, the aim of the present study was whether the analgesic effect of crocin on the chronic constriction injury (CCI) neuropathic pain could occur through interaction with the cholinergic system.

## 2. Materials and Methods

### 2.1. Animals

In the present study, 36 Wistar male rats (180 ± 20 g) were used. Animals were kept in a place with controlled temperature (20 ± 2 C), 12 hours light-dark cycles, and free access to food and water. Six rats were housed in each cage. All procedures were conducted according to National Institutes of Health Guide for Care and Use of Laboratory Animals and were approved by Ethics Committee of Semnan University of Medical Sciences and Health Services (IR.SEMUMS.RES 92/332111).

### 2.2. Drug and Dosages

Ketamine (80 mg/kg), xylazine (10 mg/kg), crocin (60 mg/kg), and atropine sulfate (0.5 and 1 mg/kg) were purchased from Sigma-Aldrich Co. Doses of drugs were selected based on previous studies [38, 39]. All drugs were used intraperitoneally. Both crocin and atropine were dissolved in physiologic saline.

### 2.3. Induction of Neuropathy

For inducing neuropathic pain, after anesthetizing (mixture of ketamine and xylazine) the rats, the upper thigh of the left leg was shaved, disinfected, and incised about 2 cm at the place of the left sciatic nerve. The exposed common sciatic nerve was separated from surrounding tissues and was ligated using four loose ligation (catgut chromic 4-0) 1 mm apart. At the end, the muscle and skin were closed using silk suture 4-0 [40]. Animals were kept in a cage individually until full consciousness was regained.

### 2.4. Experimental Protocol and Groups

**Figure 1:**
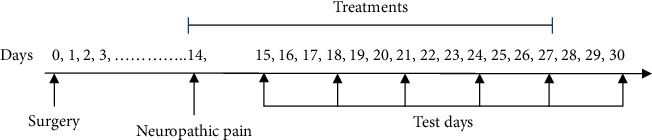
Timeline of experiments. Days 0, 1, 2, 3,…, 14, 15, 16, 17, 18, 19, 20, 21, 22, 23, 24, 25, 26, 27, 28, 29, and 30.

Animals were randomly assigned into six groups (sham, CCI, CCI + crocin60 mg/kg, CCI + atropine 0.5 mg/kg, CCI + atropine 1 mg/kg, and CCI + crocin + atropine1 mg/kg) with 6 rats in each group. Two weeks after surgery, treatment with crocin was initiated and lasted for fourteen days during which for every 3 days, behavioral tests were performed (detection of the behavioral pain response was performed 2 hours after injection). To determine if therapeutic effects of treatments were continued, 3 days following the last injection of crocin (day 30), pain behaviors were examined. Atropine sulfate was injected 30 minutes before crocin during experiments (group received atropine + crocin). The experiments were carried out according to the timeline that is shown in Figure 1.

### 2.5. Assessment of Pain Behavior of the Animals

#### 2.5.1. Mechanical Allodynia

After adapting to the environment, paw withdrawal threshold of animals was detected by Von Frey filaments [41]. Von Frey filaments, which are polyethylene hairs, are calibrated according to their diameters and each of which applies a certain amount of force (gram) to the surface. The hairs are used from low strength towards stronger one. Each filament is used three times with 10 seconds interval between each. If the animal responded to two consecutive stimulations, this number would be considered as the answer; otherwise, the experiment with the higher number would be continued. Sixty gram force was considered as the cutoff point.

#### 2.5.2. Thermal Hyperalgesia

Paw withdrawal response to thermal stimulation was detected using the plantar test device [40]. After adaptation in the plantar test device, infrared beam source was focused on the injured paw's plantar surface, and irradiation of infrared was initiated with intensity equal to 60 Hz. Latency of withdrawal response to thermal stimulation was recorded automatically. The test was performed 3 times with five minute intervals, and the average of these was considered as the paw withdrawal response. The cutoff point of test was adjusted at sixty seconds.

#### 2.5.3. Statistical Analysis

Data analysis was performed by GraphPad prism version 8.0 software (GraphPad, San Diego, CA, USA). Regarding the normal distribution of data (using the Kolmogorov–Smirnov test), two-way analysis of variance (ANOVA) was used. Sidak's multiple comparisons test was used. All statistical tests were two‐sided, and all data were expressed as mean ± S.E.M of examined variable. *P* < 0.05 was considered as the significant level.

## 3. Results

Our results showed that crocin alleviates CCI-induced neuropathic pain, and this effect was prevented using atropine pretreatment.

**Figure 2:**
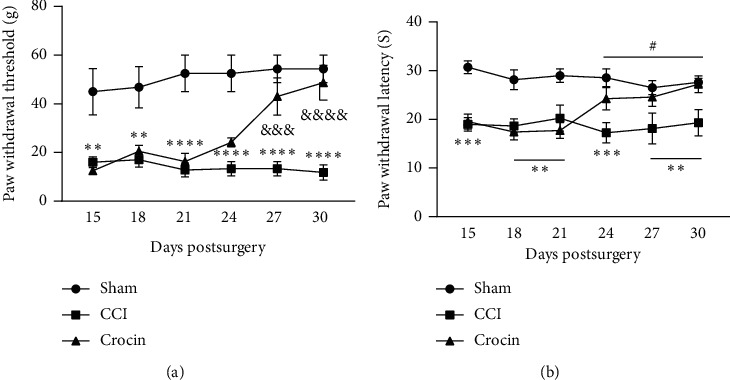
Effect of crocin on CCI-induced neuropathic pain. CCI significantly reduced mechanical (a) and thermal (b) paw withdrawal threshold compared to the sham group. Fourteen days crocin therapy significantly reduced CCI-induced mechanical allodynia and thermal hyperalgesia. Hypoalgesic effect of crocin was observed at the end of therapy (day 27) and continued on the day 30 after surgery. All data are expressed as mean ± SEM. *n* = 6. And sign and num sign compares crocin with the CCI group. Asterisks compare CCI with the sham group. #*P* < 0.05, ^*∗∗*^*P* < 0.01, ^*∗∗∗∗*^*P* < 0.0001, ^&&&^*P* < 0.01, and ^&&&&^*P* < 0.001.

Chronic constriction injury of sciatic nerve significantly reduced withdrawal threshold response to mechanical and thermal stimulus at all days examined following surgery (Figures 2(a) and 2(b)). Crocin treatment significantly prevented CCI-induced mechanical allodynia (*F*_2,90_ = 72.57, *P* < 0.0001) and thermal hyperalgesia (*F*_2,90_ = 40.09, *P* < 0.0001). After inducing neuropathic pain at day 14 after surgery, daily treatment with crocin began. Two weeks treatment with crocin significantly reduced mechanical allodynia (*P* < 0.001) and thermal hyperalgesia (*P* < 0.05). The analgesic effect of crocin continued for three days after stopping treatment (day 30 after surgery) (Figures 2(a) and 2(b)).

**Figure 3:**
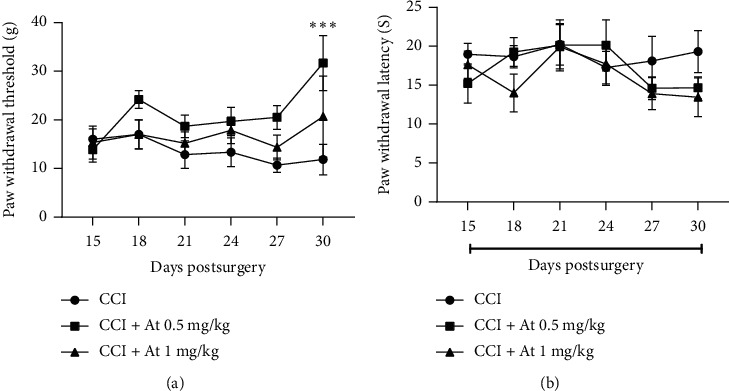
Effect of atropine on CCI-induced neuropathic pain. Effect of atropine with two doses, 0.5 and 1 mg/kg, were examined on the mechanical allodynia (a) and thermal hyperalgesia (b) during 2 weeks of treatment from 14^th^ day to 27^th^ day after surgery. Atropine with a dose of 0.5 mg/kg significantly (*P* < 0.001) reduced mechanical allodynia at day 30 after surgery (3 days after stopping treatments); however, atropine 1 mg/kg did not change CCI-induced neuropathic pain during days in which pain behaviors were examined. Asterisks compare atropine 1 mg/kg with the CCI group at day 30 after surgery. All data are expressed as mean ± SEM. *n* = 6.^*∗∗∗*^*P* < 0.001.

To determine the ineffective dose of atropine on the neuropathic pain, CCI neuropathic pain rats were subjected to atropine treatment from day 14 for fourteen days. Our results showed that atropine (0.5 mg/kg) as a muscarinic receptor antagonist could be effective on the neuropathic pain. Atropine with a dose of 0.5 mg/kg significantly (*F*_2,90_ = 7.8, *P*=0.0007) decreased mechanical allodynia (Figure 3(a)) but not thermal hyperalgesia (Figure 3(b)) at day 30 after surgery (3 days after stopping treatment). However, atropine with a dose of 1 mg/kg did not change CCI-induced mechanical allodynia and thermal hyperalgesia during the time examined. Based on this result, a dose of 1 mg/kg atropine was selected for continuing to experiment.

**Figure 4:**
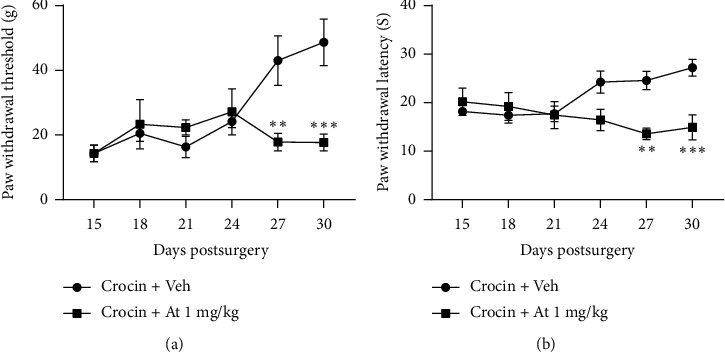
Effect of atropine pretreatment on the crocin-induced hypoalgesia. Atropine at 1 mg/kg significantly blocked the hypoalgesic effect of crocin. Fourteen days pretreatment with atropine significantly inhibited the effect of crocin on the mechanical allodynia (a) and thermal hyperalgesia (b), and these effects continued on to the 30^th^ day after surgery, while all treatments were stopped three days before. Asterisks compare atropine + crocin against that crocin group at days 27 and 30 after surgery. All data are expressed as mean ± SEM. *n* = 6. ^*∗∗*^*P* < 0.01; ^*∗∗∗*^*P* < 0.001.

According to our results, atropine with a dose of 1 mg/kg significantly prevented hypoalgesic effects of crocin. Animals received 14 days crocin therapy (i.p.) along with atropine (i.p.) pretreatment from day 14 after surgery. Our results showed that atropine significantly suppressed the improving effect of crocin on the mechanical allodynia (*F*_1,60_ = 7.266, *P*=0.009) and thermal hyperalgesia (*F*_1,60_ = 14.19, *P*=0.0004) at day 27 after surgery (Figures 4(a) and 4(b), respectively). This inhibiting effect of atropine was continued on to 30^th^ day after surgery (3 days after which treatments have been stopped).

## 4. Discussion

In the present study, crocin improved neuropathic pain induced by CCI, and atropine prevented the crocin effect.

CCI as a model for inducing neuropathic pain like behavior leads to sign and symptoms similar to human's neuropathic pain [40]. In the present research, CCI led to clear mechanical allodynia and thermal hyperalgesia (Figure 2) that reached to a peak response at 14^th^ day after surgery. This is in agreement with our previous studies that 2 weeks after surgery mechanical allodynia and thermal hyperalgesia reached to the maximum level [42].

In this study, i.p. administration of crocin during 14 consecutive days following neuropathic pain clearly showed hypoalgesia (increased paw withdrawal threshold in response to mechanical and thermal stimulation).

In agreement with present results, it has already been reported that saffron and crocin (an active constituent of saffron) significantly reduce tactile allodynia and thermal hyperalgesia in neuropathic pain rats [36]. Furthermore, previously, we showed that saffron plays a significant role in reducing neuropathic pain [43].

There is no well-known mechanism presented for the hypoalgesic effect of crocin yet, but there are several mechanisms presented for neuropathic pain so far that have a prominent role [44–48]. It has been approved that the central cholinergic system plays an important role in pain processing so that activation of central cholinoceptors lead to antinociception [45, 49–51].

Since inhibition of the muscarinic cholinergic system could be effective on neuropathic pain, in the present study, it was necessary to detect the ineffective dose of atropine on the neuropathic pain. We examined the effect of atropine on the neuropathic pain with doses of 0.5 mg/kg and 1 mg/kg and observed that mechanical allodynia and thermal hyperalgesia at day 30 following CCI (the time that treatments had been finished three days ago) significantly reduced with a dose of 0.5 mg/kg but not with a dose of 1 mg/kg (Figure 3). Depending on this result, we continued on the experiments with a dose of 1 mg/kg of atropine.

This result is in agreement with the results of other researchers that showed a dose-dependent analgesic effect for atropine [52]. They have reported that low dose (100 *μ*g/kg) of atropine through presynaptic inhibition of muscarinic autoreceptors triggers acetyl choline release which in turn increases pain threshold; meanwhile, high dose of atropine (5 mg/kg) showed a hyperalgesic effect. Therefore, in our study, it is possible that atropine with a dose of 0.5 mg/kg by triggering endogenous acetyl choline release led to increased paw withdrawal mechanical threshold. Considering the results of Ghelardini et al. [52], the administered atropine (dose of 1 mg/kg) in the present study neither is so low dose that affects as an analgesic factor nor is high dose that affects as an hyperalgesic agent. In other words, a dose of 1 mg/kg was performed as an ineffective dose on the neuropathic pain. Furthermore, it has been reported that electroacupuncture analgesia was inhibited using atropine (1 mg/kg) pretreatment in male rats [53]. Moreover, activation of spinal muscarinic M2 receptors following administration of cholinesterase inhibitors lead to analgesia [54].

We found that atropine administration before crocin inhibited the hypoalgesic effect of crocin. The present finding reveals that muscarinic receptors have a role in the effects of crocin on the neuropathic pain. This finding is in agreement with the result of other researchers that showed intrathecal administration of atropine completely inhibited electroacupuncture- (modified acupuncture technique using electrical stimulation) induced antiallodynia [55], which mediated through spinal muscarinic cholinergic receptors. Furthermore, there is a report that shows saffron significantly attenuated scopolamine-induced memory impairment [28]. Therefore, this study points out that the effect of saffron mediated through the cholinergic system. It should be mentioned that several studies have revealed that the memory improving effect of saffron is related to its active constituent crocin [56, 57]. In the present study, it would be much clearer, if we had a positive control group. However, because of financial problems, we could not provide specific agonists of muscarinic receptors or more specific antagonists to compare results, and this is one of the limitations of this study. Considering the effect of crocin on the neuropathic pain following 14 days of treatment, it is possible that crocin has led to histological or even gene expression changes that in turn led to hypoalgesia, and atropine pretreatment prevented these effects. Unfortunately, due to financial problem, we could not evaluate histological changes or gene expression following crocin therapy. However, a more detailed study can be obtained using specific agonist and antagonist or immunohistochemistry technique that results in more accurate results. We hope that we will be able to evaluate these in future research.

Regarding studies mentioned above and the present report, it seems that crocin can be suggested as a therapeutic agent against neuropathic pain, and in addition, its interaction with cholinergic antagonist's especially muscarinic must be noticed.

## 5. Conclusion

Crocin reduces CCI-induced neuropathic pain and atropine as a muscarinic antagonist blocks crocin-induced hypoalgesia. Therefore, some of hypoalgesic effects of crocin probably occur through interaction with the cholinergic system.

## Data Availability

The data used to support the findings of this study are available from the corresponding author upon request.
